# Meniscal ossicle

**DOI:** 10.4103/0971-3026.40294

**Published:** 2008-05

**Authors:** Malini A Lawande, Sidhartha Tavri, Deepak P Patkar, Sona A Pungavkar, Jayant Narang

**Affiliations:** Department of MRI, BMD and Mammography, Dr. Balabhai Nanavati Hospital, Mumbai, India

Meniscal ossicle is a very rare entity. It needs to be differentiated from a loose body as the management may differ. We present a case of meniscal ossicle, with the emphasis on MRI findings.

## Case Report

A 40-year-old man presented with chronic pain in the left knee joint for 3 years. There was no recent history of trauma. There was no other relevant past history. On clinical examination, there was mild swelling without any restriction of movements. The radiographs were unavailable but were reported to be normal.

**Figure 1:**
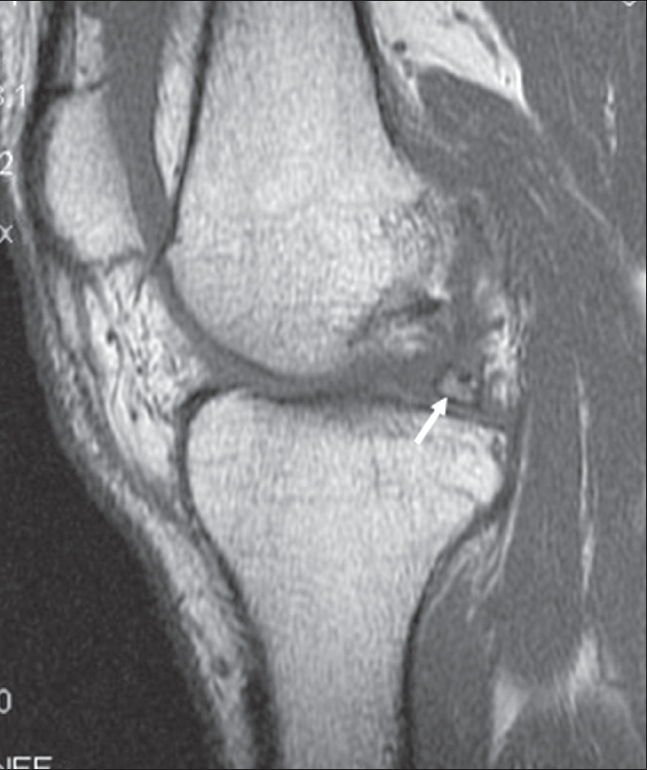
Sagittal T1W image shows a small lesion (arrow), isointense to marrow in relation to the posterior horn of the medial meniscus, with a hypointense rim

**Figure 2:**
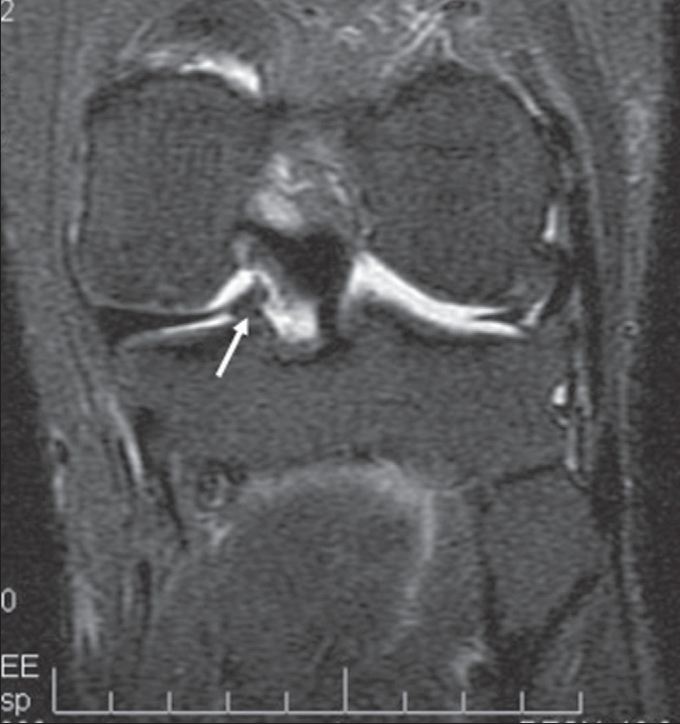
Coronal STIR image confirms the isointensity of the lesion (arrow) to the marrow

**Figure 3:**
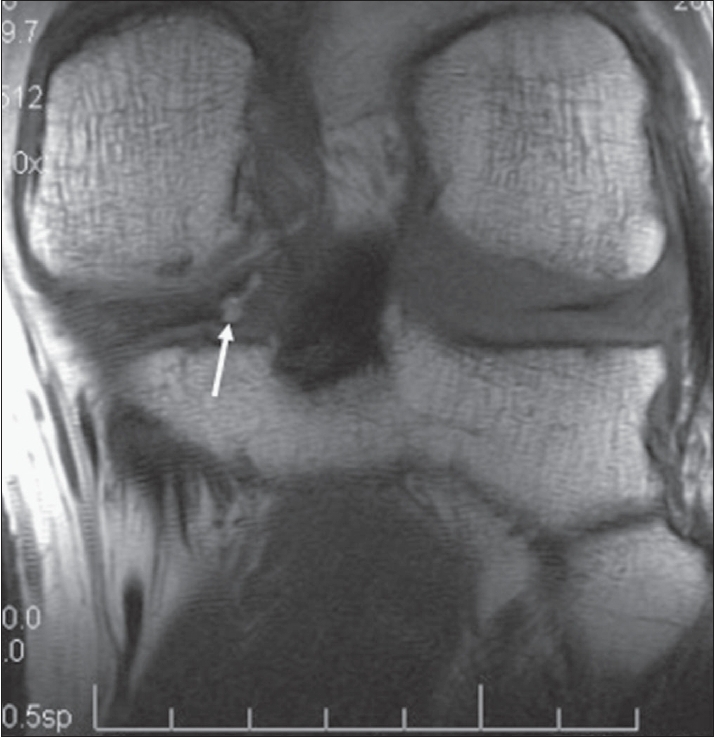
Coronal high-resolution T1W image confirms the relationship of the meniscal ossicle (arrow) to the posterior horn of the medial meniscus

MRI was performed on a 1.5T machine (GE EchoSpeed, Signa, Milwaukee, USA) using a surface coil. A well-defined lesion was seen in the medial portion of the posterior horn of the medial meniscus, isointense to bone marrow on all sequences, with a complete hypointense rim [Figures 1–3]. This was reported as a meniscal ossicle. There was myxoid degeneration in the rest of the posterior horn of the medial meniscus. Mild synovial effusion was detected. Mild changes of osteoarthritis were seen in the form of thinning of the articular cartilage and the presence osteophytes.

The patient was treated conservatively but remained symptomatic. An arthroscopy was then performed, which confirmed the finding of a meniscal ossicle, which was then removed. The patient experienced satisfactory postoperative relief.

## Discussion

Literature reports on meniscal ossicles date back to 1934, when the first case was reported by Burrows and Watson-Jones.[1] To the best of our knowledge, in these 70 odd years, it has been reported 41 times.[2] It is not clear whether this is because it is an under-diagnosed / under-reported condition or because it is actually an uncommon occurrence.

Many theories have been postulated regarding its etiology. Meniscal ossicles may be vestigial structures; they are a common occurrence in rodents, domestic cats, and in Bengal tigers.[3] An association with mucoid degeneration has been proposed,[4] but this seems unlikely since the ossicles occur more commonly in younger men, before the onset of significant mucoid degeneration.[5] A traumatic etiology has also been put forth, suggesting that the ossicles represent heterotopic ossification.[6] Alternatively, they may represent bone fragments arising from the tibial attachment of the meniscal root insertion. The last theory is supported by the fact that the most common location of meniscal ossicles is in the posterior horn of the medial meniscus,[1,5] which shows a strong attachment to the tibia and reduced mobility and is thus more prone to an avulsion tear. The normal contour of the adjoining bone on MRI however, as in this case, argues against this theory.[5] In short, there is no definite consensus on the etiology of meniscal ossicles.

Most patients complain of intermittent pain; however, since many patients also have other associated abnormalities, the relationship between the ossicles and pain is not definite.[5] A locking sensation is usually not experienced as would be expected with a free intraarticular body.[2]

Radiographically, the most common misdiagnosis is a loose body. USG in experienced hands can distinguish between loose bodies and ossicles.[7] This differentiation can also be made with arthrography and CT-arthrography, but these are invasive procedures.[7]

MRI can easily depict the ossicles located inside the substance of the meniscus,[5,8] thus distinguishing them from loose bodies, chondrocalcinosis, osteochondritis dissecans, and semimembranosus and popliteal tendon avulsions.[5] Further, their characteristic isointensity to the adjacent normal bone marrow, along with the hypointense rim, also distinguish them from loose bodies and chondrocalcinosis, the latter appearing hypointense on T1W images. The ossicles range from 7 to 10 mm in size.[5] Additionally, MRI picks up associated abnormalities such as meniscal tears, ligament tears and avulsions, cartilage damage, and synovial effusion.

The need for distinguishing between loose bodies and meniscal ossicles stems from the fact that the former require surgical intervention while the latter can be managed conservatively.[2,9] Arthroscopy is a definitive modality and arthroscopic removal of ossicles is usually the last resort, as in our case.

## References

[1] Burrows HJ (1934). Two cases of ossification in the internal semilunar cartilage. Br J Surg.

[2] Van Breuseghem I, Geusens E, Pans S, Brys P (2003). The meniscal ossicle revisited. JBR-BTR.

[3] Watson-Jones R, Roberts RE (1934). Calcification, decalcification and ossification. Br J Surg.

[4] Harris HA (1934). Calcification and ossification in the semilunar cartilages. Lancet.

[5] Schnarkowski P, Tirman PF, Fuchigami KD, Crues JV, Butler MC, Genant HK (1995). Meniscal ossicle: Radiographic and MR imaging findings. Radiology.

[6] Berg EE (1991). The meniscal ossicle: The consequence of meniscal avulsion. Arthroscopy.

[7] Martinoli C, Bianchi S, Spadola L, Garcia J (2000). Multimodality imaging assessment of meniscal ossicle. Skeletal Radiol.

[8] Tuite MJ, De Smet AA, Shannon Swan, Keene JS (1995). MR imaging of a meniscal ossicle. Skeletal Radiol.

[9] Glass RS, Barnes WM, Kells DU, Thomas S, Campbell C (1975). Ossicles of knee menisci: Report of seven cases. Clin Orthop Relat Res.

